# IL-17 and IL-22 production in HIV+ individuals with latent and active tuberculosis

**DOI:** 10.1186/s12879-018-3236-0

**Published:** 2018-07-11

**Authors:** Kamakshi Prudhula Devalraju, Venkata Sanjeev Kumar Neela, Sharadambal Sunder Ramaseri, Arunabala Chaudhury, Abhinav Van, Siva Sai Krovvidi, Ramakrishna Vankayalapati, Vijaya Lakshmi Valluri

**Affiliations:** 1 Immunology & Molecular Biology Department, Bhagwan Mahavir Medical Research Centre, A. C. Guards, Hyderabad, TS 500004 India; 2 Clinical Division, Cheyutha, LEPRA Society, Cherlapally, Hyderabad, 501301 India; 3 Department of Pulmonary Immunology, Centre for Biomedical Research, University of Texas Health Centre, 11937 US Highway 271, Tyler, TX 75708 USA; 4 Department of Biotechnology, Sreenidhi Institute of Science and Technology, Yamnampet, Ghatkesar, Hyderabad, Telangana-501301 India

**Keywords:** Human, Latent tuberculosis, HIV, Cytokines, IL-22, IL-17

## Abstract

**Background:**

IL-17 and IL-22 cytokines play an important role in protective immune responses against *Mycobacterium tuberculosis* (Mtb) infection. Information on the production of these cytokines and the factors that regulate their production in the context of human immunodeficiency virus (HIV) and latent tuberculosis infection (LTBI) or active tuberculosis disease (ATB) is limited. In the current study, we compared the production of these two cytokines by PBMC of HIV-LTBI+ and HIV + LTBI+ individuals in response to Mtb antigens CFP-10 (culture filtrate protein) and ESAT-6 (Early Secretory Antigenic Target). We also determined the mechanisms involved in their production.

**Methods:**

We cultured Peripheral Blood Mononuclear Cells (PBMCs) from HIV- individuals and HIV+ patients with latent tuberculosis and active disease with CFP-10 and ESAT-6. Production of IL-17, IL-22 and PD1 (Programmed Death 1), ICOS (Inducible T-cell Costimulator), IL-23R and FoxP3 (Forkhead box P3) expression on CD4+ T cells was measured.

**Results:**

In response to Mtb antigens CFP-10 and ESAT-6, freshly isolated PBMCs from HIV+ LTBI+ and HIV+ active TB patients produced less IL-17 and IL-22 and more IL-10, expressed less IL-23R, and more PD1 and expanded to more FoxP3+ cells. Active TB infection in HIV+ individuals further inhibited antigen specific IL-17 and IL-22 production compared to those with LTBI. Neutralization of PD1 restored IL-23R expression, IL-17 and IL-22 levels and lowered IL-10 production and reduced expansion of FoxP3 T cells.

**Conclusions:**

In the current study we found that increased PD1 expression in HIV + LTBI+ and HIV+ active TB patients inhibits IL-17, IL-22 production and IL-23R expression in response to Mtb antigens CFP-10 and ESAT-6.

**Electronic supplementary material:**

The online version of this article (10.1186/s12879-018-3236-0) contains supplementary material, which is available to authorized users.

## Background

*Mycobacterium tuberculosis* (Mtb) infects one-third of the world’s population and causes almost 1.3 million deaths per year [1, 2]. Approximately 90% of infected persons develop latent tuberculosis infection (LTBI), and remain well, but 10% develop primary tuberculosis (TB) soon after infection or reactivation TB many years later [3]. HIV infection markedly increases susceptibility to TB, and HIV-infected persons with LTBI have an 800-fold greater risk of developing active TB (www.cdc.gov/tb/). TB is the leading cause of death in HIV-infected persons and more than half a million co-infected people die annually (www.avert.org/tuberculosis.htm).

Pro-inflammatory Th17 cytokines (IL-17A, IL-17F, IL-21 and IL-22) are important in conferring protection against Mtb infection [4]. IL-17, released by antigen-experienced CD4 cells [5] is critical in vaccine-induced protective immune responses against Mtb infection [4, 6, 7] We demonstrated earlier that reduced IL-17 production by CD4+ T cells of tuberculosis patients was associated with decreased IL-23R and increased PD1 expression by CD4+ T cells [8]. IL-22 produced by human NK cells, inhibits intracellular growth of Mtb [9]. In a mouse model, IL-22 decreases the number of immunosuppressive T-regulatory cells and contributes to the efficacy of BCG vaccination, decreasing the bacillary burden and increasing antigen-specific T-cell responses after challenge with Mtb [10].

In the current study, we tested the hypothesis whether increased PD1 expression in HIV + LTBI+ individuals enhances FoxP3+ cell expansion and IL-10 production, inhibits the expression of IL-23R and production of IL-17 and IL-22 in response to Mtb antigens CFP-10 and ESAT-6.

## Methods

### Patient population

Forty patients with acid-fast smear, and culture-confirmed active pulmonary tuberculosis (ATB), with no history of anti-tuberculosis therapy seropositive to HIV were enrolled. Forty HIV patients with latent TB infection with no history of ART/ ATT were enrolled. These patients attended the Integrated Counseling and Testing Centre (ICTC) and outpatient clinics under LEPRA Society Hyderabad, India. HIV infection was confirmed by Combs (Arkray healthcare Pvt.Ltd), followed by Trispot and SD bioline tests. Thirty five LTBI- individuals with and without HIV infection were also enrolled as a control group for some experiments. Written informed consent was obtained from all participants before enrolling in the study.

#### Characteristics

HIV patients with CD4 counts above 350 cells/mm^3^ attending the ICTC clinics were counseled and enrolled depending on the CD4 counts. Age, BCG history and years of onset of HIV infection were obtained. HIV + TB patients in our cohort were newly diagnosed to both HIV and TB. (Table 1).

**Table 1:** Demographic and clinical characteristics of study subjects

	HIV-LTBI-	HIV-LTBI+	HIV + LTBI-	HIV + LTBI+	HIV + TB+
Number of participants	35	40	35	40	40
Mean age	34.4 ± 6.9	31.7 ± 7.1	32.4 ± 8.3	33.1 ± 6.5	34.6 ± 6.2
Percentages of males and females	51, 49	55,45	43, 57	56,44	61,39
BCG scar percentage	90%	85%	70%	68%	73%
History of TB infection	No	No	No	No	No
Mean CD4 counts	747 ± 356	869 ± 408.9	408 ± 307.1	525 ± 323.3	250 ± 259
Mean CD8 counts	836 ± 420.3	728 ± 334.3	1187 ± 606.7	1227 ± 913.5	663 ± 501.6
Mean years of onset of HIV infection	NA	NA	9	10	NA
ART treatment	NA	NA	No	No	No

*NA* = Not Applicable, *HIV-LTBI*-: HIV negative healthy individuals without latent *TB* infection, *HIV-LTBI*+: *HIV* negative healthy individuals with latent *TB* infection. *HIV + LTBI*-: HIV patients without latent TB, *HIV + LTBI*+: HIV patients with latent *TB, HIV + TB+: HIV* patients with active tuberculosis

#### Inclusion criteria

ART naïve HIV patients with CD4+ cell counts> 350 were enrolled. HIV+ active TB patients naïve to ART or ATT were enrolled irrespective of their CD4 counts.

#### Exclusion criteria

HIV+ individuals with past history of active TB and any other opportunistic infections like HCV or HERPES and those on ART and with other lifestyle diseases like diabetes were excluded. Pregnant women also were excluded from the study.

The study was approved by the Institutional Ethical Committee of Blue Peter Public Health Research Centre, Hyderabad, India**.**

### Antigens for stimulation assays

For stimulation of PBMC we used ESAT-6 and CFP-10 peptide pools (BEI resources) consisting of 21 and 22 peptides covering the entire 6-kDa ESAT-6 and 10-kDa CFP-10, respectively*.*

### Antibodies and other reagents

FITC anti-CD4, PE anti-FoxP3 and PE and APC anti- ICOS (all BD Biosciences) and IL-23R (R and D systems) were used. To determine the absolute CD4 and CD8 counts BD Trucount tubes and BD Multitest antibodies were used. For neutralization experiments, we used anti- PD1, and isotype control antibodies (Bio legend).

### Determination of absolute CD4 and CD8 counts

Absolute CD4 and CD8 counts in the blood were determined by flow-cytometry (FACS CALIBUR, BD Biosciences) using MultiTEST four-color antibodies and TruCount tubes. MultiSET software was used for analysis (BD Biosciences).

### Isolation and culture of PBMCs

Freshly isolated PBMCs were cultured in 24-well plates at 2 × 10^6^ cells/well in RPMI 1640 containing 1% penicillin/streptomycin (Sigma), L-Glutamine and 10% heat-inactivated human serum, with or without γ-*Mtb* CFP-10 + ESAT-6 (10 μg/ml) at 37 °C in a humidified 5% CO_2_ atmosphere. To neutralize PD1, 10 μg/ml anti-PD1 antibody (BioLegend) was added to the cells in presence of the antigen. Isotype control antibody μg/ml (BioLegend) was added to some cells this served as a control well for PD1 specific inhibition. After 96 h, cell-free culture supernatants were collected, aliquoted and stored at − 70 °C until cytokine concentrations were measured by ELISA as published in [8]. Cells were washed and stained for surface and intracellular markers.

### Flow cytometry

Percentages of PD-1, ICOS, and IL-23R and FoxP3-positive CD4 cells were determined by flow cytometry. Both freshly isolated and cultured PBMC, were incubated with each of the antibodies in respective tubes along with anti-CD4 at 4 °C for 30 mins in dark. To determine FoxP3 population we used the intracellular staining kit from eBiosciences. For surface staining, FITC anti-CD4 and APC anti-CD25 were added. After washing with PBS and 2% FCS, cells were fixed in 1× fixation/ permeabilization/ buffer and washed twice in 1× permeabilization/wash solution. Anti-FoxP3 PE was then added to cells re-suspended in staining buffer. After incubation at 4 °C for 30 min, cells were washed in PBS with 2% FCS, and analyzed by flow cytometry.

### Gating

From the total lymphocyte population CD4+ and CD8+ T cells were identified by their respective fluorochrome tags. PD1, ICOS and IL-23R were gated on CD4+ cells and percentage of double positive population was determined based on the quadrant set by an isotype control antibody. (Additional file 1: Figs. S1, S2, S3, S4).

### Determination of latency in the study subjects

PBMCs (2 × 10^6^) were stimulated with and without 10 μg/mL of CFP-10 and ESAT-6 pooled peptide antigens and incubated for 96 h. IFN-γ levels in culture supernatants were measured by sandwich ELISA using commercial human interferon-gamma kit (eBioscience Inc., San Diego, CA, USA) following manufacturer’s instructions. Absorbance at 450 nm was subtracted from the value at 540 nm. The concentrations were calculated using MPM software version 6.1. The unstimulated wells served as negative controls, the IFN-γ values from unstimulated supernatants were used to determine the “cut-off” value to identify LTBI. The cutoff was calculated as the (mean + 2*SD) of IFN-γ concentration all the unstimulated wells. IFN-γ concentration above the cut-off value was considered positive for LTBI. ROC curve was plotted with IFN-γ values and data was analyzed using SPSS 16.0 software.

### Measurement of IFN-γ, IL-17A, IL-22 and IL-10 concentrations

IFN-γ, IL-17A, IL-22 and IL-10 in culture supernatants were determined by ELISA (eBiosciences) following manufacturer’s instructions.

### Statistical analysis

Results are shown as mean ± SE. For data that were normally distributed, comparisons between groups were performed by a paired or unpaired *t* test, as appropriate. For data that were not normally distributed, the non-parametric Mann-U-Whitney test was performed.

Multivariate Analysis of Variance (MANOVA), was used to analyze cytokine secretion, CD4 cell counts and PD1 expression. The effects of these variables and the interaction between study groups was analyzed by the Pillai’s Trace Multivariate test using SPSS package 16.0. The correlation coefficients were determined using Graph pad prism v5.0 software.

## Results

### Specificity and sensitivity of in house interferon gamma release assay (IGRA)

**Fig. 1:**
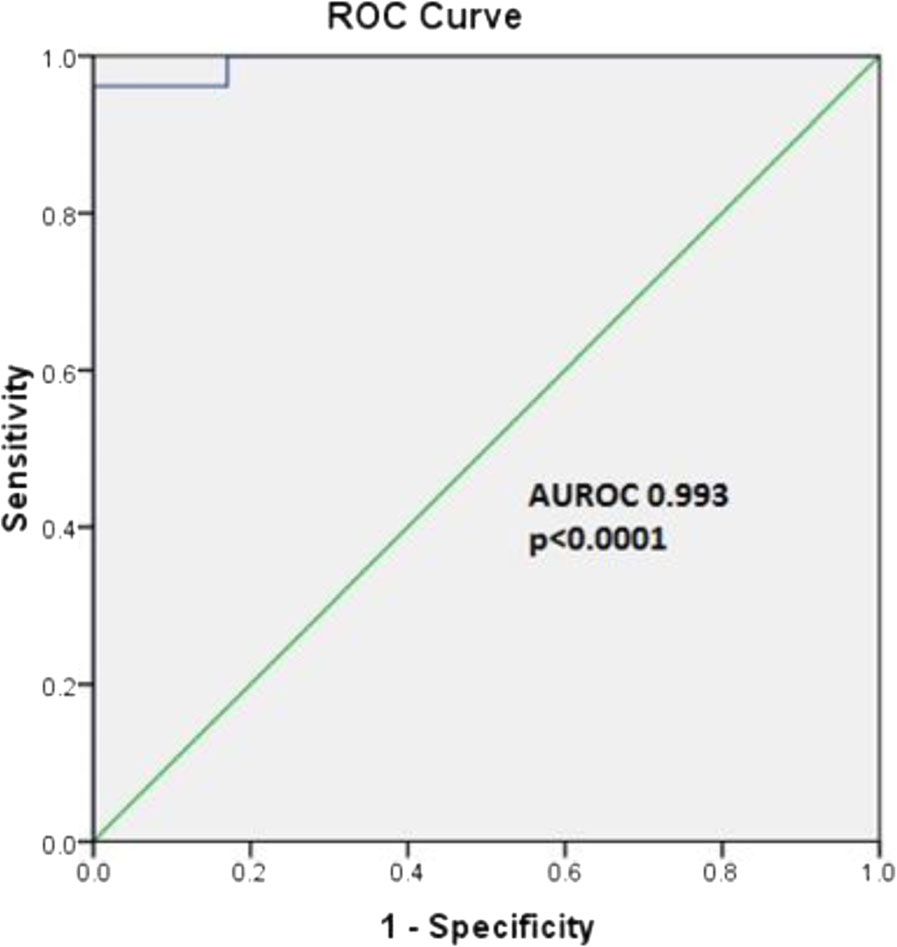
Receiver operating characteristic (ROC) curve for IFN-γ values obtained from HIV- and HIV+ individuals with or without LTBI. LTBI status as determined by the inhouse assay was compared to commercially available QGT. Area under the ROC curve (AUC) 0.993 (95% CI = 0.98 to 1.0), *p* < 0.0001

Our In-house IGRA was 96.7% specific and 95% sensitive compared to conventional QuantiFERON –TB Gold (QFT-G) kit as demonstrated in the Receiver Operating Characteristic curve (ROC) (Fig. 1).

### Cytokine production by antigen specific stimulated PBMCs

**Fig. 2:**
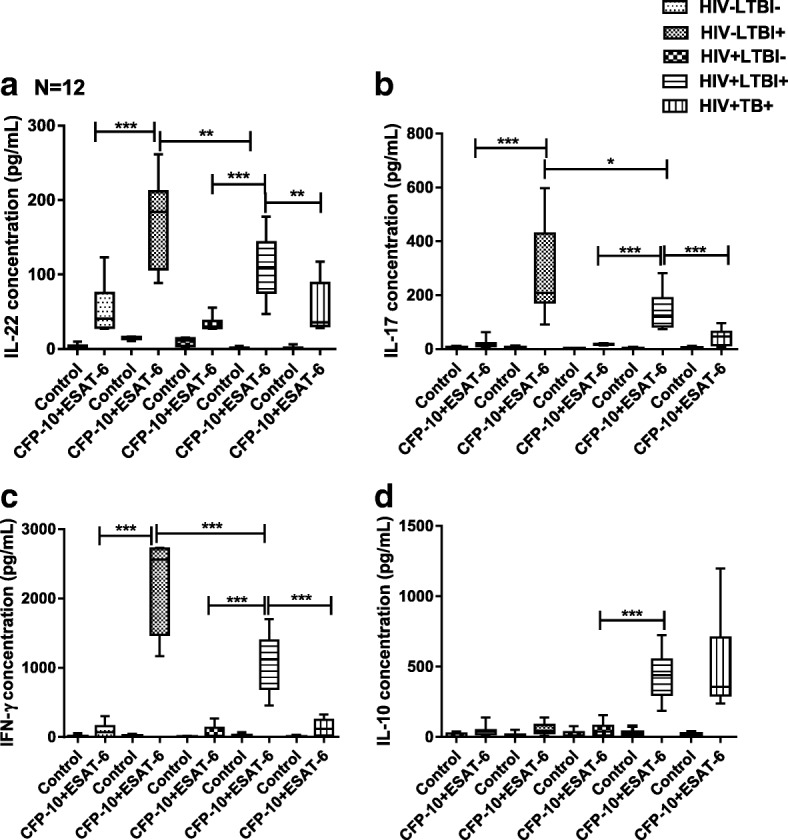
Mean values of cytokine levels in stimulated PBMC culture supernatants. Freshly isolated PBMC (12 donors in each group) from HIV-LTBI-, HIV-LTBI+, HIV + LTBI-, HIV + LTBI+ individuals, and HIV + TB+ patients were cultured with or without ESAT-6 and CFP-10 (10 μg/ml each). After 96 h, cytokine levels in culture supernatants were determined by ELISA. Bars show (**a**). IL-22, (**b**). IL-17, (**c**) IFN-γ, (**d**). IL-10 levels in culture supernatants

CFP-10 and ESAT6 significantly enhanced IL-22 (*p* = 0.064), IL-17 (*p* = 0.0184) and IFN-γ (*p* < 0.0001) production by freshly isolated PBMC of healthy HIV-LTBI+ individuals compared to that of HIV + LTBI+ patients (Fig. 2a, b, c). Active TB disease further decreased IL-22 (*p* = 0.0013), IL-17 (*p* < 0.0001) and IFN-γ (*p* < 0.0001) production by CFP-10 and ESAT6 stimulated PBMC of HIV + LTBI+ individuals (Fig. 2a, b, c). In contrast, CFP-10 and ESAT6 significantly enhanced IL-10 production by PBMCs of HIV + LTBI+ (*p* < 0.0001) compared to healthy HIV-LTBI+ individuals (Fig. 2d).

HIV-LTBI- controls produced significantly less IL-22 (*p* = 0.064), IL-17(p = 0.0184) and IFN-γ (*p* < 0.0001) compared HIV-LTBI+ individuals, and HIV-LTBI- patients produced significantly less IL-22 (*p* = 0.064), IL-17 (*p* = 0.0184) and IFN-γ (p < 0.0001) compared to that of HIV + LTBI+ patients (Fig. 2a, b, c).

### PD-1 expression by T-cells

**Fig. 3:**
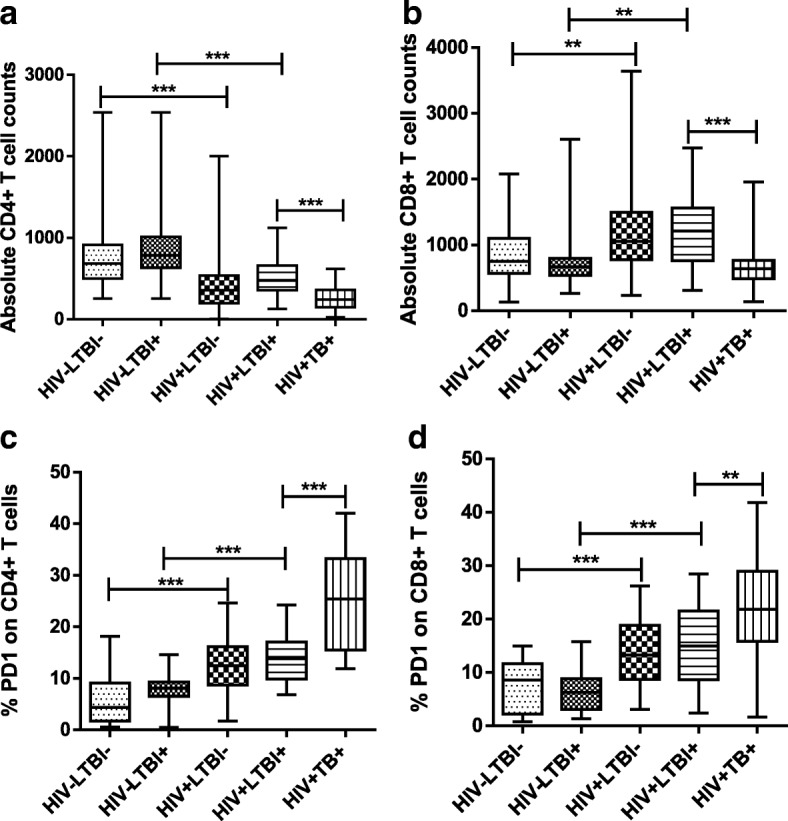
Mean values of CD4+ and CD8+ T-cell absolute counts and PD1 expression **(a**, **b**) Absolute CD4+ and CD8+ Tcells in blood**.** Whole blood from HIV-LTBI-, HIV-LTBI+, HIV + LTBI-, HIV + LTBI+ individuals, and HIV + TB+ patients (29 donors in each group) was collected. Four color staining (CD3, CD4, CD8 and CD45) was performed using TruCount tubes and data was analysed using MultiSET software. (**c**, **d**) PD-1 expression on CD4+ and CD8+ T-cells. Freshly isolated PBMC (21 donors in each group) from HIV-LTBI-, HIV-LTBI+, HIV + LTBI-, HIV + LTBI+ individuals, and HIV + TB+ patients were collected. The percentages of CD4 + PD-1+ and CD8 + PD-1+ cells were determined by flow cytometry. Data are shown as the median (horizontal line), 25th and 75th percentile values (box), and 5th and 95th percentile values (whiskers)

The absolute numbers of CD4+ (*p* < 0.0001) but not CD8+ (*p* = 0.0028) cells in freshly isolated PBMCs were higher in healthy HIV-LTBI+ individuals than in HIV + LTBI+ patients (Fig. 3a, b). Active TB disease further decreased the absolute number of CD4+ (p < 0.0001) and CD8+ (p < 0.0001) cells in freshly isolated PBMCs HIV+ individuals (Fig. 3a, b). PD-1 expression by CD4+ (p < 0.0001) and CD8+ (p < 0.0001) cells of freshly isolated PBMC was higher in HIV + LTBI+ patients compared to healthy HIV-LTBI+ individuals (Fig. 3c, d). Active TB disease further increased the PD-1 expression on CD4+ (p < 0.0001) and CD8+ (*p* = 0.0071) cells of HIV+ individuals (Fig. 3c, d).

Similar to LTBI+ individuals CD4+ (p < 0.0001) but not CD8+ (*p* = 0.004) cells in freshly isolated PBMCs were higher in healthy HIV-LTBI- individuals than in HIV + LTBI- patients (Fig. 3a, b). PD-1 expression by CD4+ (p < 0.0001) and CD8+ (p < 0.0001) cells of freshly isolated PBMC was higher in HIV + LTBI- patients compared to healthy HIV-LTBI- individuals (Fig. 3c, d).

### ICOS, IL-23R and FoxP3 expression by freshly isolated CD4+ cells

**Fig. 4:**
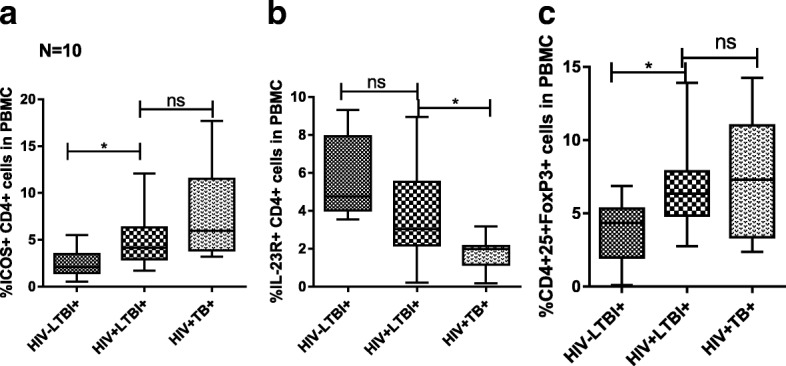
Mean values of ICOS, IL-23R and FoxP3 expression by CD4+ cells Freshly isolated PBMC (10 donors in each group) from HIV-LTBI+, HIV + LTBI+ individuals and HIV + TB patients were collected. The percentage (**a**) CD4 + ICOS+ (**b**) CD4 + IL-23R+ and (**c**) CD4 + CD25 + FoxP3+ cells were determined by flow cytometry

ICOS (*p* = 0.04) and FoxP3 (*p* = 0.0203) expression was higher in HIV + LTBI+ patients compared to healthy HIV-LTBI+ individuals (Fig. 4a, c). In contrast, there was no difference in IL-23R expression in HIV + LTBI+ patients compared to that of healthy HIV-LTBI+ individuals (Fig. 4b). There was no difference in ICOS and FoxP3 expression of HIV + LTBI+ and HIV + TB patients (Fig. 4a, c). However, IL-23R expression in HIV + TB patients was significantly decreased when compared to HIV + LTBI+ individuals (*p* = 0.0284) (Fig. 4b).

### Anti-PD-1 antibodies enhance IL-17, IL-22, IFN-γ and decrease IL-10 production by HIV + LTBI+ individuals and HIV + TB patients

**Fig. 5:**
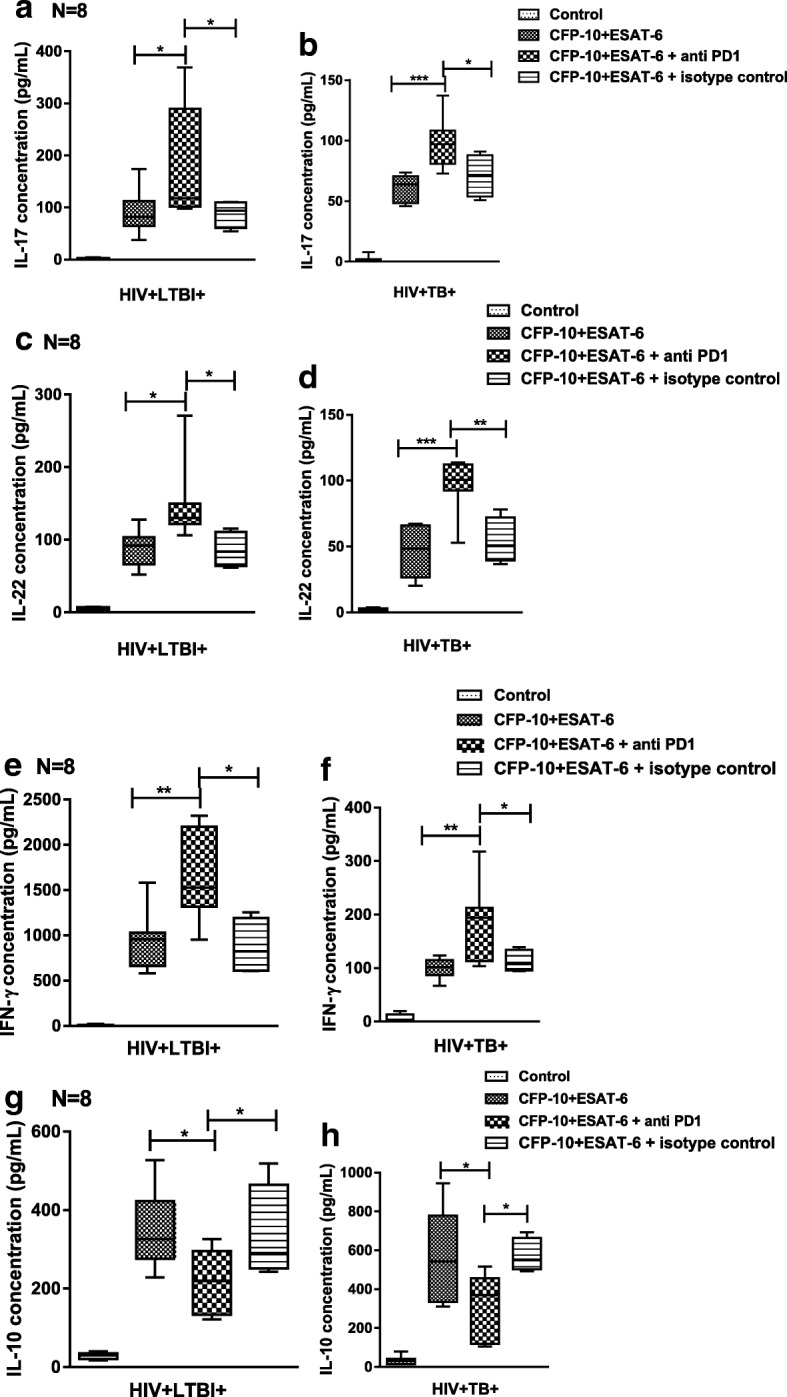
Mean cytokine values of cytokine levels after PD1 neutralization Freshly isolated PBMC (8 donors in each group) from HIV-LTBI+, HIV + LTBI+ individuals, and HIV + TB patients were cultured with or without ESAT6 and CFP10 (10 μg/ml each) and in the presence of anti-PD-1 or isotype control antibodies. After 96 h, cytokine levels in culture supernatants were determined by ELISA. Bars show (**a**, **b**) IL-17, (**c**, **d**) IL-22, (**e**, **f**) IFN-γ, (**g**, **h**) IL-10 levels in culture supernatants

In HIV+ individuals with either latent or active TB, anti-PD-1 antibodies significantly enhanced CFP-10 and ESAT6 induced IL-17 (*p* = 0.047 & 0.0008), (Fig. 5a, b); IL-22 (*p* = 0.025 & 0.0006), (Fig. 5c, d); IFN-γ (*p* = 0.0088 & 0.0174), (Fig. 5e, f); but decreased IL-10 significantly (*p* = 0.0163 & 0.0261) (Fig. 5g, h); compared to CFP-10 and ESAT6 alone.

Anti-PD1 antibodies significantly enhanced IL-17 (*p* = 0.05&0.01), (Fig. 5a, b); IL-22 (p = 0.04 & 0.004), (Fig. 5c, d); IFN-γ (*p* = 0.031 & 0.021), (Fig. 5e, f); but decreased IL-10 significantly (p = 0.04 & 0.03) (Fig. 5g, h) compared to isotype control antibody.

### Anti-PD-1 antibodies improve antigen specific IL-23R and ICOS expression and inhibit FoxP3 expression by HIV + LTBI+ individuals and HIV + TB patients

**Fig. 6:**
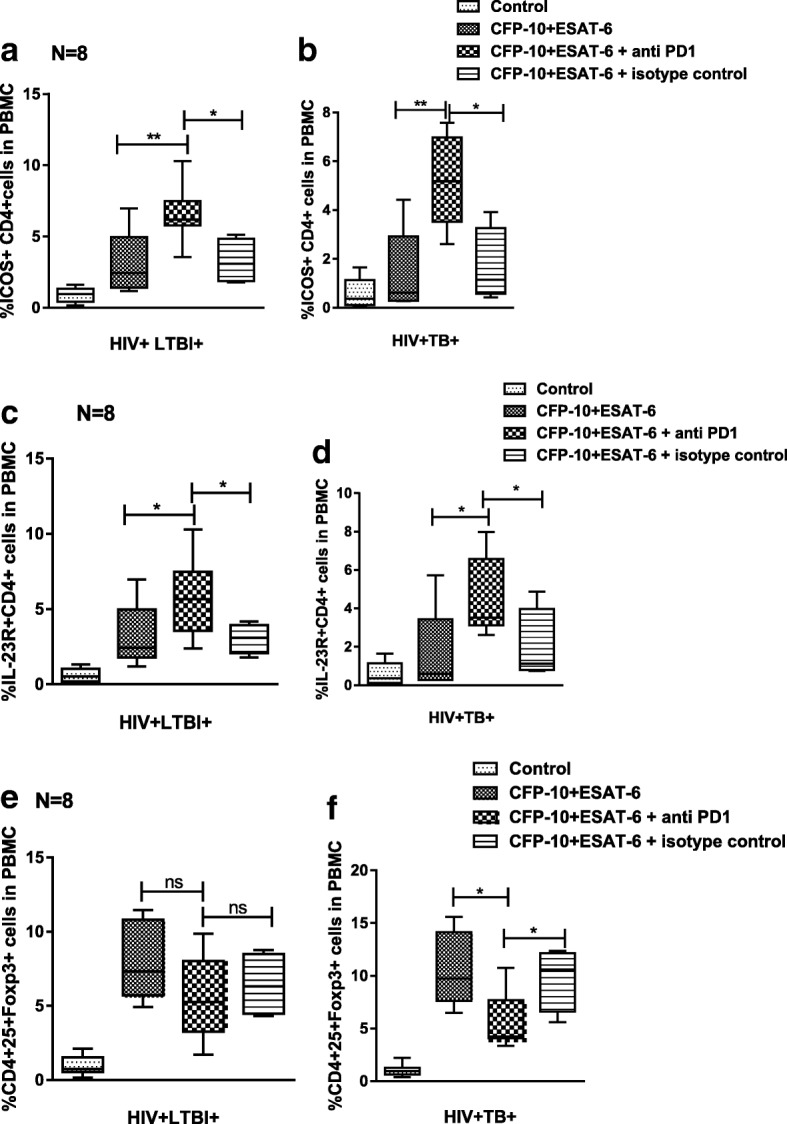
Mean values of IL-23R, ICOS and FoxP3 expression on CD4 + T cells after PD-1 neutralization. Freshly isolated PBMC (8 donors in each group) from HIV + LTBI+ individuals, and HIV + TB patients were cultured with or without ESAT6 and CFP10 (10 μg/ml each) and in the presence of anti-PD-1 or isotype control antibodies. After 96 h, the percentages CD4 + ICOS+ (**a**, **b**) CD4 + IL-23R+ (**c**, **d**), and CD4 + FoxP3+ (**e**, **f**) cells were determined by flow cytometry. Boxes show the median and interquartile range, and whiskers show the 5th and 95th percentile values

In HIV+ individuals with either latent or active TB, anti-PD-1 antibodies significantly enhanced CFP-10 and ESAT6 induced ICOS (*p* = 0.0090 & 0.0026), (Fig. 6a, b); IL-23R (*p* = 0.0452 & 0.037), (Fig. 6c, d); expression but decreased FoxP3 expression only in HIV + TB+ patients (*p* = 0.0167) (Fig. 6f) compared to CFP-10 and ESAT6 alone.

Anti-PD-1 antibodies significantly enhanced CFP-10 and ESAT6 induced ICOS (p = 0.01 & 0.03), (Fig. 6a, b); IL-23R (p = 0.03 & 0.042), (Fig. 6c, d); expression but decreased FoxP3 expression only in HIV + TB+ patients (p = 0.04) (Fig. 6f) compared to isotype control antibody.

### MANOVA to determine the effect of the variables on HIV, LTBI and ATB

The Pillai’s trace multivariate statistic in MANOVA test indicated that there was an overall significant effect of cytokine expression, absolute CD4 counts and PD1 expression on the study groups (F = 8.47, *p* < 0.001, Pillai’s trace Λ value = 2.13, partial η2 = 0.53). (Table 2).

**Table 2:** MANOVA test between subjects and effects

Source	Dependent Variable	F	Significance	Partial Eta Squared	Observed Power^a^
Study Groups	IL-22	26.68	< 0.001	0.66	1
	IL-17	22.65	< 0.001	0.622	1
	IFN-γ	88.04	< 0.001	0.865	1
	IL-10	25.39	< 0.001	0.649	1
	CD4 Counts	29.95	< 0.001	0.685	1
	PD1 on CD8	15.52	< 0.001	0.53	1
	PD1 on CD4	32.32	< 0.001	0.702	1

^a^Computed using alpha = 0.05

Results of MANOVA test for calculation of significant variances

The analysis also revealed a significant global effect of study groups (*F* = 8.47, *p* < 0.001) on cytokine level, CD4 counts and PD1 expression with significance group differences in IL-22 (F = 26.7, p < 0.001), IL-17 (F = 22.6, p < 0.001), IFN-γ (F = 88.0, p < 0.001), IL-10 (F = 25.4, p < 0.001), absolute CD4 counts (F = 29.9, p < 0.001), PD1 expression on CD4 (F = 15.5, p < 0.001) and CD8 cells (F = 232.3, p < 0.001).

## Discussion

Recent studies demonstrated that Th17 cytokines, IL-17 and IL-22 play an important role in protective immune responses against Mtb infection [11]. During HIV infection, IL-17 has both protective and pathological roles [12–14] but IL-22 has a protective role [15]. HIV infection depletes mucosal IL-22-producing T-cell subsets during very early stages of infection [16]. This is evident in Simian immunodeficiency virus (SIV) infection since loss of these cells correlates with disease progression, immune activation, and viral persistence [17, 18]. However, there is no information available about IL-17 and IL-22 production and factors that regulate their production in HIV infected individuals with latent and active tuberculosis infection.

We found that PBMC from HIV-LTBI- and HIV-LTBI+ individuals have similar number of PD1 + CD4 and PD1 + CD8 cells. But in response to Mtb antigens CFP-10 and ESAT-6, PBMC from LTBI+ individuals produced higher amounts of IL-17 and IL-22 compared to LTBI- individuals suggesting antigen specific memory T-cells are major producers of these two cytokines. We also found that in response to Mtb antigens CFP-10 and ESAT-6, PBMC from HIV + LTBI+ and HIV+ active TB patients produce less amounts of these two cytokines compared to HIV-LTBI+ individuals. As a control, we also measured IFN-γ and IL-10 levels in the above culture supernatants. Our studies are in agreement with previous findings [19, 20] that CFP-10 and ESAT-6 cultured PBMC from HIV + LTBI+ and HIV+ active TB patients produced less IFN-γ and more IL-10. Our findings further demonstrate that in response Mtb antigens CFP-10 and ESAT-6, PBMC from HIV + LTBI+ and HIV + active TB patients produce less of two important protective Th17 cytokines IL-17 and IL-22 compared to HIV-LTBI+ healthy individuals.

In our previous studies, we found that increased PD1 expression is responsible for reduced IL-23R expression and IL-17 production in TB patients [8]. We also found anti-tuberculosis therapy [ATT] reduced PD1 expression on CD4+ T cells and restored IL-23R expression and IL-17 production [8]. Our current findings demonstrates that HIV infection enhances PD1 expression by T cells during Mtb infection. Neutralization of PD-1 enhanced IL-17, IL-22, IFN-γ production and reduced IL-10 production by CFP-10 and ESAT-6 cultured PBMC of HIV + LTBI+ and HIV+ active TB patients. HIV is known to enhance PD1 expression [21, 22] causes viral specific CD4 and CD8 T cell exhaustion and loss of effector functions and failure to control viral replication [23, 24]. Our findings further demonstrates that enhanced PD1 expression in HIV + LTBI+ and HIV+ active TB patients reduces protective Th17 cytokine production.

It is known that ICOS and FoxP3+ T-regulatory cells are known to regulate IL-17 and IL-22 production. ICOS is a T-cell activation marker and known to be associated with increase in viral activity [25, 26] and inhibits IL-22 production [27]. Tregs (CD4 + CD25 + FoxP3+ cells) are essential for maintenance of peripheral tolerance and homeostasis, and most studies show that Tregs inhibit immunity and worsen disease due to pathogenic organisms [28]. PD1 and cytokine inducible SH2-containing protein (CISH) control the expansion of CD4^+^CD25^+^FoxP3^+^ cells during Mtb infection [29, 30]. Increased number of CD4^+^FoxP3^+^ T-cells in TB patients were found to inhibit immune responses [29, 31, 32]. FoxP3+ cells suppress IL-17 [33] and IL-22 [34] production by T cells through IL-10 production. We found increased number of FoxP3+ cells in HIV + LTBI+ and HIV+ TB patients compared to HIV-LTBI+ individuals. IL-23R is essential for IL-17 production [35] and in the current study, we found decreased IL-17 and IL-22 production is associated with reduced IL-23R expression by CD4+ cells of HIV + LTBI+ and HIV + active TB patients. Overall, our current findings suggest that increased PDI expression reduces expression of IL-23R and ICOS and increases expansion of Treg which leads to reduced IL-17 and IL-22 production.

## Conclusions

In conclusion we found enhanced PD1 expression by T cells reduces IL-17 and IL-22 production in HIV + LTBI+ and HIV+ active TB patients in response to Mtb antigens CFP-10 and ESAT-6. Our observations suggest anti-PD1 antibody and recombinant IL-22 can be used as immune therapeutic agents to prevent the development of active TB in HIV + LTBI+ individuals.

## Additional file

Additional file 1: **Figure S1.** PD1 expression on CD4+ and CD8+ T cells. Freshly isolated PBMCs were stained with antibodies to PD1, CD4 and CD8. PD1 expression on (a) CD4 and (b) CD8 positive T cells was determined by flow cytometry. Plot shows percentages of (c) CD4 + PD1+ and (d) CD8 + PD1+ T cells in a HIV+ patient. Figure S2 IL-23R expression by CD4+ T cells. Freshly isolated PBMCs were stained with antibodies to CD4 and IL-23R. Plot shows a. CD4 positive cells in lymphocytes. b. CD4 isotype control antibody. (c). CD4 + IL-23R+ cells in healthy controls. CD4+ IL-23R+ cells in CFP-10 + ESAT-6 stimulated PBMCs (d, f) before and (e, g) after blocking PD1 in HIV + LTBI+ and HIV + TB+ patients respectively. Figure S3 FoxP3 expression by CD4+ T cells. Freshly isolated PBMCs were stained with antibodies to CD4, CD25 and FoxP3. Plot shows a. CD4 isotype control antibody. (b). CD4 + CD25 + FoxP3 cells in healthy controls. CD4 + CD25 + FoxP3 cells in CFP-10 + ESAT-6 stimulated PBMCs (c, e) before and (d, f) after blocking PD1 in HIV + LTBI+ and HIV + TB+ patients respectively Figure S4 ICOS expression by CD4+ T cells. Freshly isolated PBMCs were stained with antibodies to CD4 and ICOS. Plot shows a. CD4 isotype control antibody. (b). CD4 + ICOS+ cells in healthy controls. CD4 + ICOS+ cells in CFP-10 + ESAT-6 stimulated PBMCs (c, e) before and (d, f) after blocking PD1 in HIV + LTBI+ and HIV + TB+ patients respectively. (PPTX 201 kb)

## Abbreviations

AFB: Acid fast bacilli
APC: Allophycocyanin
ATT: Anti Tuberculosis Therapy
BCG: Bacillus Calmette Guerin
CD: Cluster of differentiation
CFP-10: Culture Filtrate Protein
ELISA: Enzyme Linked Immuno-Sorbent Assay
ESAT: Early Secreted Antigenic Target
FCS: Fetal calf serum
FITC: Fluorescein isothiocyanate
FoxP3: Forkhead box P3
HIV: Human immunodeficiency virus
ICOS: Inducible T-cell Co-stimulator
IFN: Interferon
IL: Interleukin
LTBI: Latent Tuberculosis Infection
Mtb: *Mycobacterium tuberculosis*
NK: Natural killer
PBMC: Peripheral Blood Mononuclear Cells
PBS: Phosphate Buffered Saline
PD1: Programmed Death 1
PE: Phycoerythrin
SD: Standard deviation
SE: Standard error
SIV: Simian Immunodeficiency Virus
T cells: T-Lymphocytes
TB: Tuberculosis
TGF: Transforming Growth Factor
