# Supplementary material for: IL-17 and IL-22 production in HIV+ individuals with latent and active tuberculosis
Source: BMC Infect Dis. 2018 Jul 11;18:321. doi: 10.1186/s12879-018-3236-0 (PMC6042451; doi:10.1186/s12879-018-3236-0)
Supplement: Supplementary file 1 — Figure S1. PD1 expression on CD4+ and CD8+ T cells. Freshly isolated PBMCs were stained with antibodies to PD1, CD4 and CD8. PD1 expression on (a) CD4 and (b) CD8 positive T cells was determined by flow cytometry. Plot shows percentages of (c) CD4 + PD1+ and (d) CD8 + PD1+ T cells in a HIV+ patient. Figure S2 IL-23R expression by CD4+ T cells. Freshly isolated PBMCs were stained with antibodies to CD4 and IL-23R. Plot shows a. CD4 positive cells in lymphocytes. b. CD4 isotype control antibody. (c). CD4 + IL-23R+ cells in healthy controls. CD4+ IL-23R+ cells in CFP-10 + ESAT-6 stimulated PBMCs (d, f) before and (e, g) after blocking PD1 in HIV + LTBI+ and HIV + TB+ patients respectively. Figure S3 FoxP3 expression by CD4+ T cells. Freshly isolated PBMCs were stained with antibodies to CD4, CD25 and FoxP3. Plot shows a. CD4 isotype control antibody. (b). CD4 + CD25 + FoxP3 cells in healthy controls. CD4 + CD25 + FoxP3 cells in CFP-10 + ESAT-6 stimulated PBMCs (c, e) before and (d, f) after blocking PD1 in HIV + LTBI+ and HIV + TB+ patients respectively Figure S4 ICOS expression by CD4+ T cells. Freshly isolated PBMCs were stained with antibodies to CD4 and ICOS. Plot shows a. CD4 isotype control antibody. (b). CD4 + ICOS+ cells in healthy controls. CD4 + ICOS+ cells in CFP-10 + ESAT-6 stimulated PBMCs (c, e) before and (d, f) after blocking PD1 in HIV + LTBI+ and HIV + TB+ patients respectively. (PPTX 201 kb) [file 12879_2018_3236_MOESM1_ESM.pptx]

## Slide 1
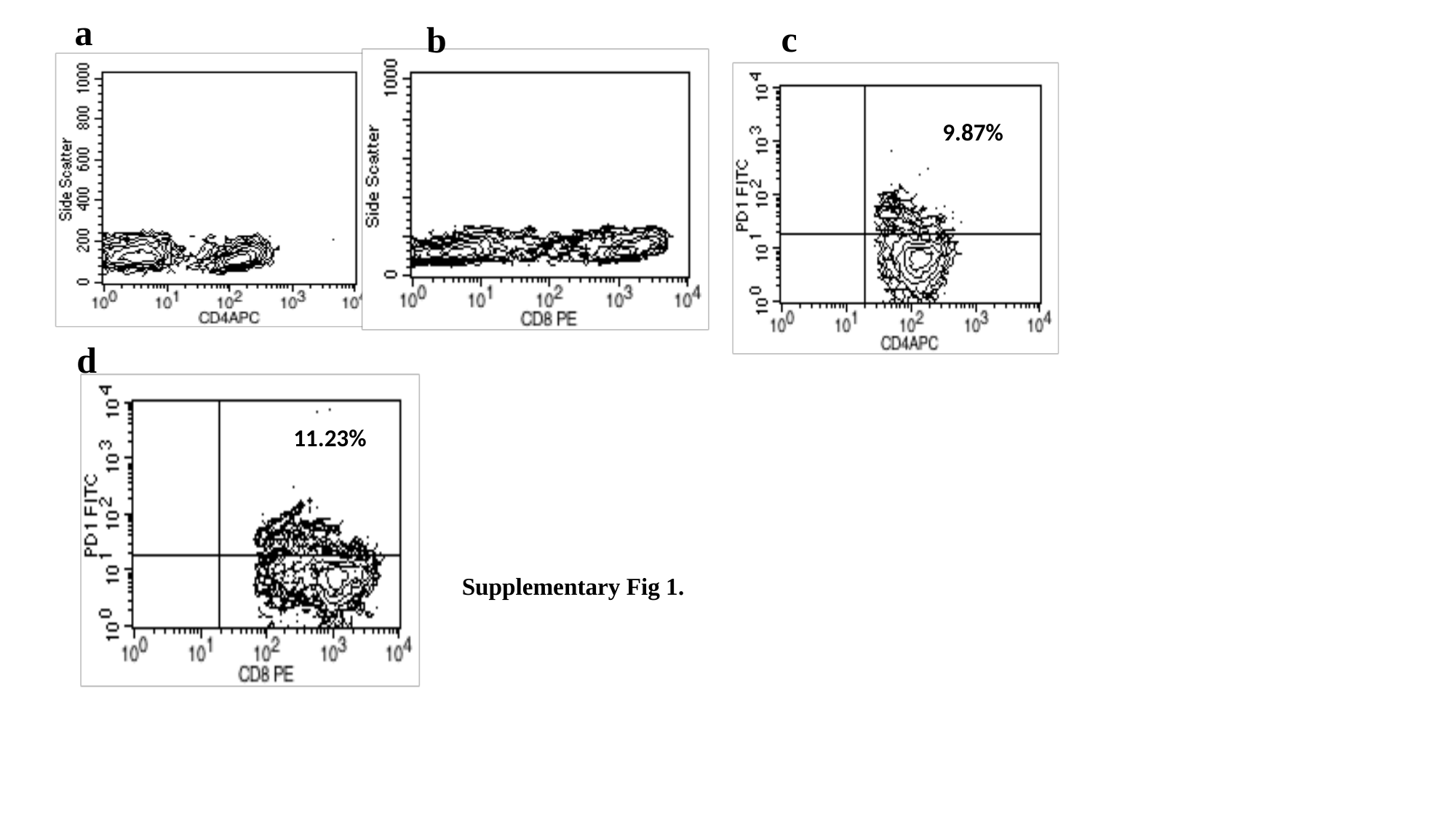

a
c
b
9.87%
d
11.23%
Supplementary Fig 1.

## Slide 2
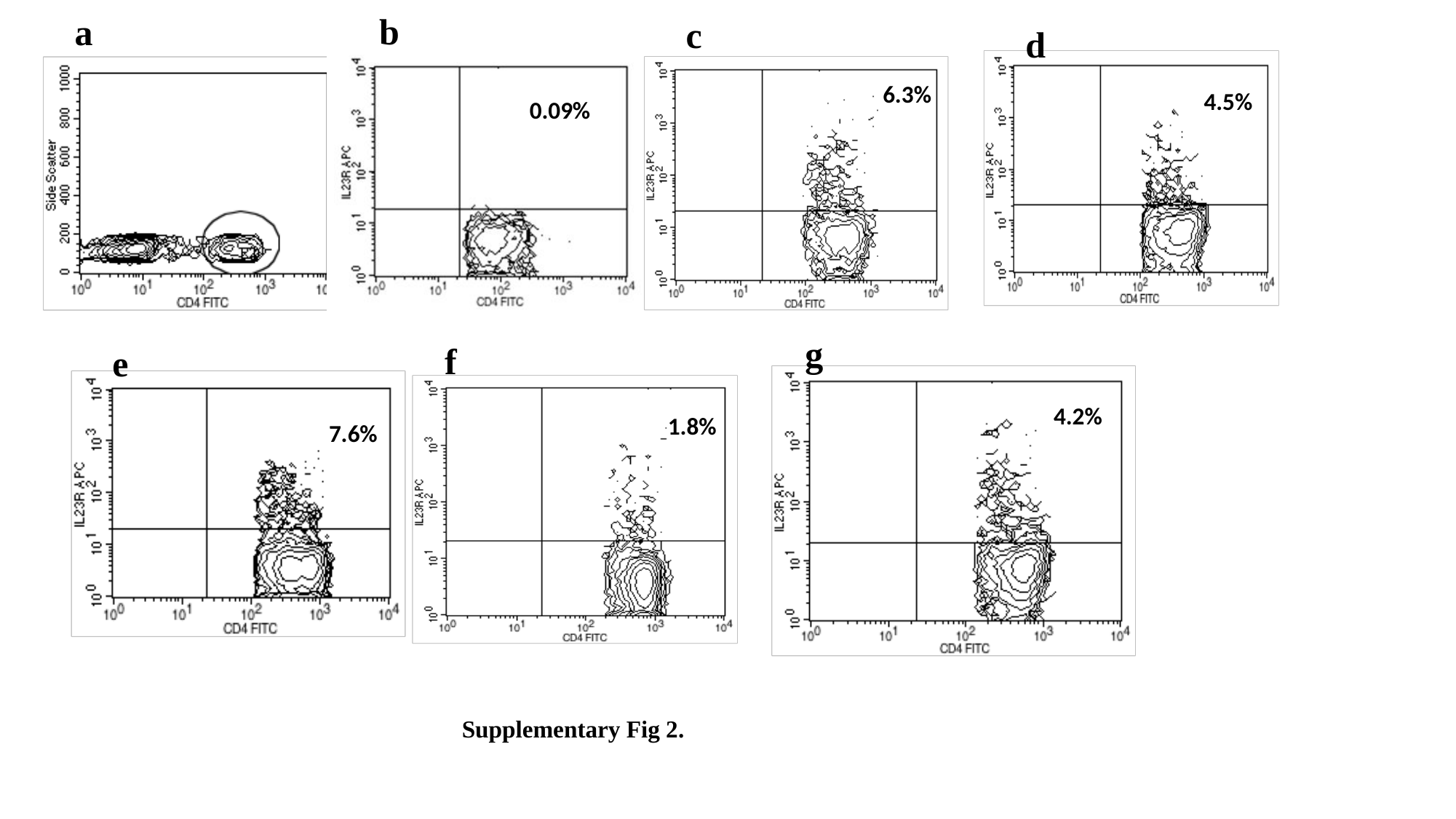

b
a
c
d
6.3%
4.5%
0.09%
g
f
e
4.2%
1.8%
7.6%
Supplementary Fig 2.

## Slide 3
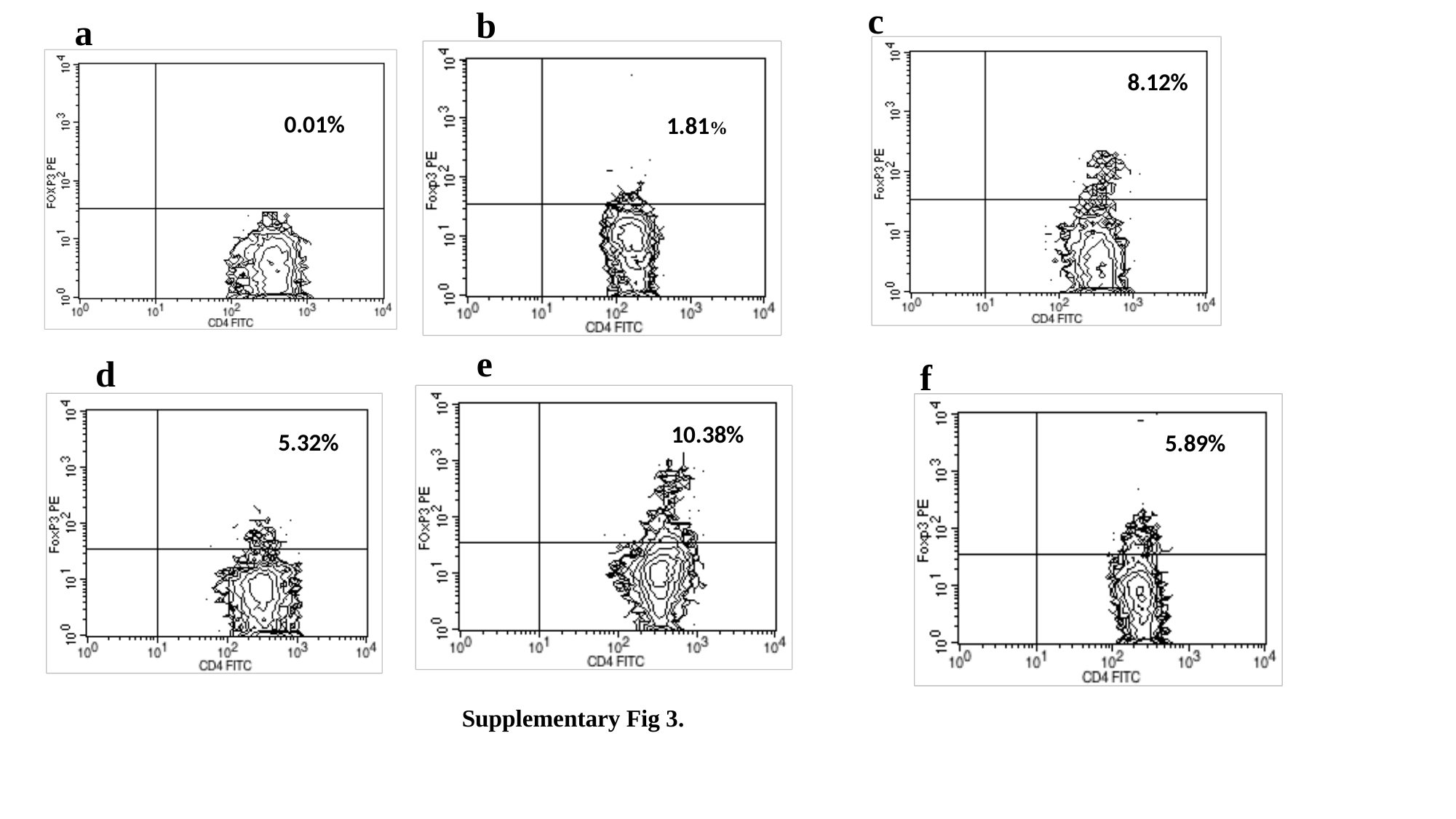

c
b
a
8.12%
0.01%
1.81%
e
d
f
10.38%
5.32%
5.89%
Supplementary Fig 3.

## Slide 4
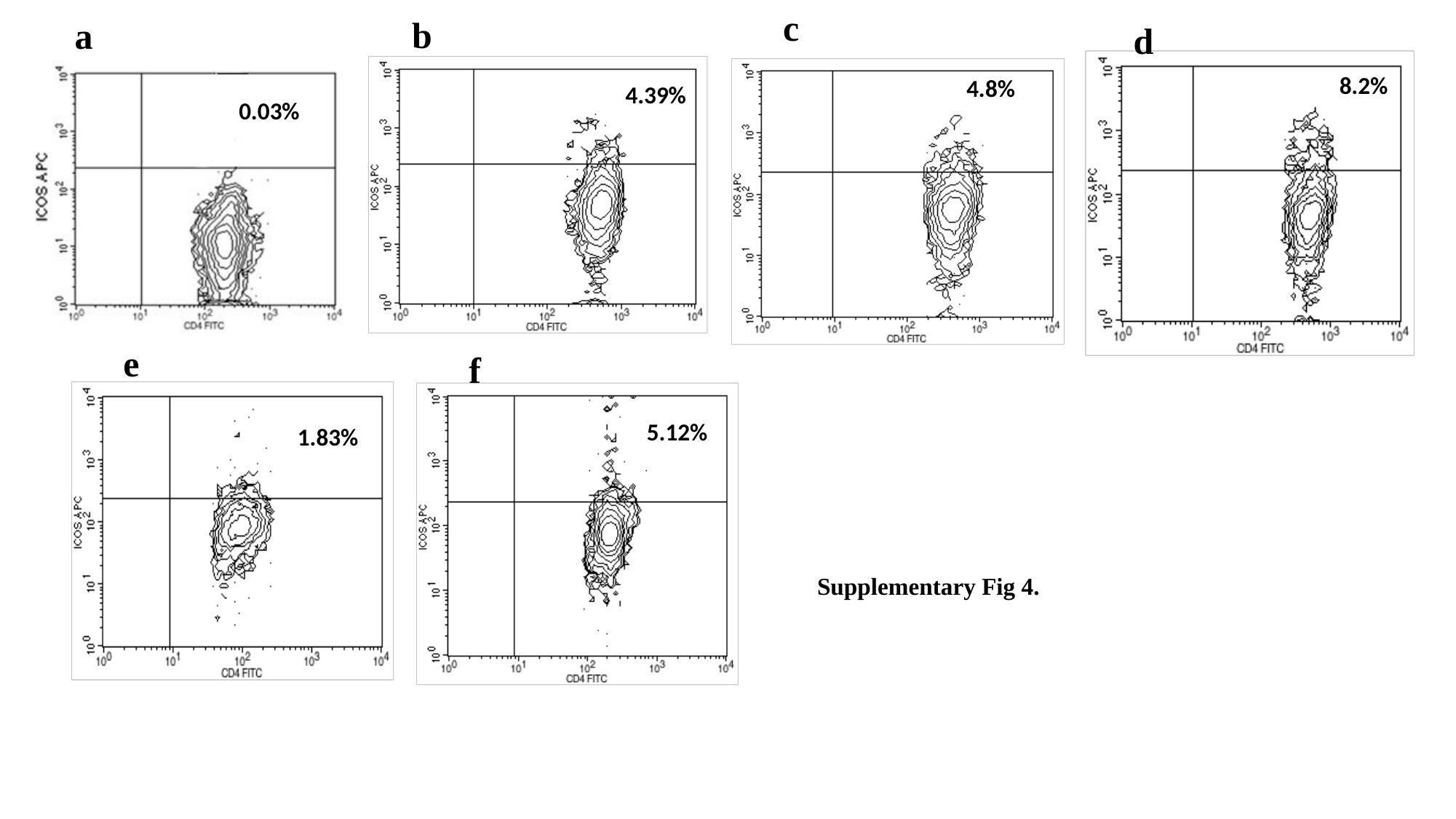

c
b
a
d
8.2%
4.8%
4.39%
0.03%
0.03%
e
f
5.12%
1.83%
Supplementary Fig 4.
